# Working in harmony with Nature: highlights from 2014, and a look to the future

**DOI:** 10.1038/npjpcrm.2015.31

**Published:** 2015-04-23

**Authors:** Paul Stephenson, Aziz Sheikh

**Affiliations:** 1 Editors-in-Chief

‘In all things of nature there is something of the marvellous.’ Aristotle^2^

A year has now elapsed since the *Primary Care Respiratory Journal (PCRJ)* was re-launched as *npj Primary Care Respiratory Medicine* published by Nature Publishing Group (NPG). In so doing it became the first of the Nature Partner Journals, a major open access publishing initiative from NPG (http://www.nature.com/content/NPJ/index.html). The publishing agreement established between NPG and the Primary Care Respiratory Society UK (PCRS-UK), in conjunction with the International Primary Care Respiratory Group (IPCRG), was, of course, a landmark for this journal*—*and in our editorial in March 2014 we expressed our delight at the announcement and our belief that the journal would benefit hugely from being published by a top international academic publisher.^3^ And so it has turned out to be. With apologies to both Epictetus and Aristotle, this last year has provided many opportunities for working ‘in harmony with Nature’^1^ and there really has been ‘something of the marvellous’^2^ as we have developed our relationship with the team at NPG… One year on, we thought it appropriate to review the events of 2014, to expand on some of the highlights, and to give you a flavour of our plans for the future.

## Online-only and open access: dramatic increase in website visits

There were two major changes which emanated from the new publishing partnership. The first was the loss of the issue-based print version of the journal. All Nature Partner Journals are online-only, fully open access journals and are hosted on the nature.com web platform. All *npj Primary Care Respiratory Medicine* articles are therefore freely available on our journal website (www.nature.com/npjpcrm) and as default are published under the CC-BY Creative Commons licence, which provides the widest possible opportunities for re-use whilst authors retain copyright. New content is published as soon as it is ready for publication rather than waiting to be assigned to an issue.

Though we understand that the loss of the print issue was a disappointment for some, for PCRS-UK members this gap has now been filled by the new society publication ‘Primary Care Respiratory Update’. However, most of our readers, particularly international readers, have for years read the journal online. The increase in traffic to the new journal website over the last 10 months has been quite extraordinary: there has been a 9% increase in page views and a 14% increase in site visitors *month on month*, culminating in 15,441 site visits and 19,483 page views/month by January of this year. These figures comfortably exceed those from previous years (site visits: 8,204/month in 2013).

## An article processing charge

The second change was the introduction of an Article Processing Charge (APC), to be paid by authors upon article acceptance*—*an integral part of open access publishing. The trend in academic journal publishing is very much towards open access, with some research funders (e.g. the Wellcome Trust in the UK) now mandating publication of research in fully open access journals, and an increasing number of open access journals for authors to choose from.

Our APC currently stands at £2,650 ($4,000, 3,300 Euros) for original research articles, and £1,000 ($1,500, 1,250 Euros) for all other article types (such as Review articles, Perspectives and Brief communications)*—*as is the case for all Nature Partner Journals. Understandably, authors will ask what they are getting in return for their APC… One of the main advantages of being hosted on the nature.com platform is that all authors who publish in *npj Primary Care Respiratory Medicine* benefit from maximum dissemination of content through one of the most prestigious scientific web platforms in the world. The dramatic increase in website visits over the last 10 months means that authors’ work is getting increased exposure and is being brought to the attention of a much wider audience than was previously the case; our readership is expanding all the time and so this is a benefit that will increase incrementally. Detailed article metrics are available for every published article, with daily updates on page views and social media activity (for example, see http://www.nature.com/articles/npjpcrm20149/metrics). Our marketing team use online banner adverts across the nature.com platform as well as e-mail campaigns and social media adverts to highlight authors’ work, and our ‘Featured article’ is placed prominently at the top of the website homepage (www.nature.com/npjpcrm).

All of this means that article submission rates have remained strong and that authors are continuing to send us high quality manuscripts. We are delighted that this is the case.

## Open access publication: seeking financial assistance

Despite all the advantages of open access publishing, we fully understand that payment of an APC can be an obstacle to some. However, we are adamant that authors should not feel inhibited about submitting manuscripts to us simply because of the APC, especially if they consider our journal the ideal setting for their work. We as Editors, NPG our publisher, the PCRS-UK and the IPCRG are in complete agreement on this. Information about research funders and institutions that provide funding for open access is available from the NPG open access funding page (http://www.nature.com/authors/open_access/funding.html). In addition, the journal operates an APC waiver policy for authors from HINARI countries, and NPG will consider applications for the APC to be sponsored in instances where authors cannot pay the full amount for publication (http://www.nature.com/npjpcrm/open-access).

## Correspondence

A glance at our Guide for authors (http://www.nature.com/authorguide/npjpcrm/npjpcrm-gta.pdf) shows that one type of article does *not* require payment of an APC: correspondence. As we stated in our first editorial as newly-appointed Editors-in-Chief in 2011, ‘critical discussion and feedback are the bread-and-butter of scientific progress, and we are very keen to see this encouraged’.^4^ We reiterate our belief in this sentiment, and therefore we’re delighted to receive correspondence relating to any article published in the journal within six weeks of that article being published. Once accepted for publication, correspondence will be published free of charge. Of course, the more correspondence submissions we receive, the more selective we will have to be in what we publish! For now, however, we encourage readers to put pen to paper (or, more correctly, finger to keyboard…), and in so doing you have a reasonably good expectation that your contribution will be published.

## Impact factor

However much we might proclaim the advantages of the nature.com platform in disseminating authors’ work, a journal’s Impact factor is of crucial importance for authors planning to submit their work for publication. Our Impact factor jumped to 2.909 this year, up from 2.191 the year before, meaning that the journal is now ranked 2/18 in the Primary Health Care category and 21/53 in the Respiratory System category. Our strategic aim is to increase our Impact factor to > 5.0 over the next few years, and so this is encouraging. However, given that our citable article Impact factor denominator is only about 60/year, it’s likely that there will be some to-ing and fro-ing on the journey! We will continue to be highly selective in choosing only the very best articles for publication.

## Publishing the best: thanks to our editors and reviewers

Choosing the best articles, of course, involves high quality peer review, and we are proud to hear from various sources that the journal has developed a reputation for the quality of its peer review over the years. This is in large part due to our superb Associate Editors who handle the vast majority of manuscripts, but it’s also due to our reviewers (who almost invariably provide peer review of the highest quality) and our Editorial Board members (who regularly review papers and give of their time and expert advice frequently). Every quantitative paper undergoes a thorough statistical review by Gopal Netuveli, our Statistical editor. In addition, our Education section editors Hilary Pinnock and Jaime Correia de Sousa regularly commission high quality articles which are particularly relevant to practising clinicians in the primary care setting; in so doing, we are fulfilling the second of the journal’s two aims (http://www.nature.com/npjpcrm/about/aims).

We thank all of our editors, reviewers and Editorial Board members for their expertise and support: it’s a team effort, and we really are extremely grateful. Our tribute to the full list of 2014 reviewers was published recently on the journal website (http://www.nature.com/public/article-assets/npg/npjpcrm/reviewers/2014reviewers.pdf).

## Manuscript handling times

In 2014 our median time to first decision for original research articles was 21 days, and for all other article types it was 19 days. Median time to final decision for original research articles was 36 days, and for all other article types it was 23 days. These handling times are highly competitive, and we’re justifiably proud of them. Occasionally there will be the odd paper where there has been an unforeseen delay in obtaining expert reviews, but authors can expect their manuscript to be dealt with scrupulously fairly in an efficient and timely manner. For those manuscripts which we feel do not warrant peer review, authors will receive a rejection decision within a week of submission*—*which at least gives them a speedy response and a chance to select another journal for submission quickly. Our manuscript handling times have improved year on year, and we are delighted to pay tribute to our excellent NPG Editorial office team for their speed and efficiency.

## Healthy competition

The increase in our Impact factor this year, our reputation for high quality peer review and our quick manuscript handling times do indeed make this journal ‘highly competitive’. But we entirely agree with the *European Respiratory Journal (ERJ)* editors^5^ that healthy competition amongst leading journals is to be welcomed since it reflects the dynamism in respiratory research. As they highlighted in their recent editorial, there is indeed a collegiate spirit in respiratory publishing. The respiratory journal editors meet twice a year to analyse developments and to discuss the challenges common to all medical journal editors, and there is always a mutually supportive atmosphere during these discussions. There have been times over the last 18 years when the support of our respiratory editor colleagues has been invaluable to the editors (past and present) of this journal, and for this we are very grateful indeed.

## Respiratory medicine in primary care: article highlights in 2014

*npj Primary Care Respiratory Medicine* has its feet firmly in two camps: respiratory medicine, and primary care. The journal publishes work from clinicians and academics working within any sector that impacts on the primary care management of respiratory and respiratory-related allergic diseases.

It seems invidious to highlight some papers as opposed to others, but in 2014 there were a number of papers that excelled, either in terms of website page views, or in terms of the social media activity and correspondence they generated. In asthma, we published a case-control study on its association with obesity in children,^6^ and one of the largest ever European surveys of patients’ symptoms and experience of the disease.^7^ There were also a number of papers on various aspects of pharmacological management.^8,9^

In chronic obstructive pulmonary disease (COPD), we published comparable papers on epidemiology, management and outcomes from the UK and Sweden,^10,11^ a paper on specialist nurse versus primary care diagnosis of COPD,^12^ a randomised controlled trial (RCT) of tiotropium in patients naïve to maintenance treatment,^13^ an RCT of a cognitive-behavioural manual versus information leaflets in terms of their effect on health status,^14^ and a paper from the PLATINO study on the criteria for selecting patients for spirometry.^15^ We do publish a number of qualitative papers, and two in particular focussed on patients’ experience of identifying and managing COPD exacerbations,^16^ and coordination of end-of-life care.^17^

Outside of asthma and COPD, there were notable papers on inappropriate prescribing of inhaled corticosteroids for respiratory tract infections,^18^ the diagnosis of interstitial lung disease,^19^ and the safety of domiciliary intravenous antibiotics in bronchiectasis.^20^

## A look to the future

There are a number of projects that are coming to fruition over the next few months. The first is the migration of the old *PCRJ* archive to the nature.com platform, which was completed last month. This important development means that readers will have access on the nature.com platform to all articles published by this journal since 1997 to the present day.

The second is the development of our online pre-submission enquiry facility. As soon as this is established, if authors send in an abstract we will give them a rapid preliminary assessment within 5 working days telling them whether we’re interested in a submission; authors will then know whether or not it’s worth formatting their work according to our Guide for authors (http://www.nature.com/authorguide/npjpcrm/npjpcrm-gta.pdf). In the meantime, please don’t hesitate to contact the Editorial office at npjpcrm@nature.com.

The third is the development of the journal website (www.nature.com/npjpcrm). In fact, this is an ongoing project, and the NPG web development team are constantly looking at ways to develop the journal website, making it even more attractive and helping readers navigate and discover relevant content. There will be increased use of photos and graphics to accompany each published article, and we may even consider a photographic competition with a prize for the best photo submitted by authors...

Fourthly, we are in discussion with NPG and other Nature editors so that authors will automatically be offered a number of alternative in-house submission options if their respiratory manuscript has been rejected by one of the NPG journals. Once these details have been finalised, we will let you know.

One of the features of the old *PCRJ* was that we regularly published translations of selected articles to aid accessibility for readers in countries such as Spain, Portugal and Brazil. The IPCRG and NPG are currently in discussion about this, and the ambition remains to restart a translation service for selected articles as soon as possible. Again, we will keep you updated...

Finally, in association with the NPG Press office and our marketing team, we will be developing our online social media and general media strategy in order to increase the profile of selected papers even more.

We think this all sounds exciting and we hope you do too. It certainly bodes well for the future.

## Working in harmony with nature

Joe Bennett is the Senior Publishing Manager for the Nature Partner Journals at NPG, and since we were the first of the ‘npj’ journals we’ve had the privilege of working very closely with him over this last year or more; we are indebted to him for all his hard work, advice and encouragement. Martin Delahunty is the Global Head of Partnership Journals for NPG, and we thank him wholeheartedly for his support. We’ve already thanked our Editorial office team, but we must also thank our Production team (in particular Sue Bigmore) for their scrupulous attention to detail, as well as Emma Hedington and the marketing team for their success in aiding the dissemination of authors’ work and for spreading the news about *npj Primary Care Respiratory Medicine*.

We are also really grateful for the support we’ve received from the PCRS-UK Trustees (particularly Patrick White) and the Executive Committee, Anne Smith the PCRS-UK Chief Executive, and also the IPCRG Board and its Executive Officer Sian Williams.

It truly does require a team effort to publish an academic journal such as this, and we are privileged to be part of it. 2014 was a really exciting year for the journal, with its transformation into *npj Primary Care Respiratory Medicine* and a new publishing partner in NPG. We have every confidence that 2015 will prove to be equally good. Our final thanks, of course, must go to you, all of our contributors and readers, without whom there would be no journal...
